# The Involvement of Neuroinflammation and Kynurenine Pathway in Parkinson's Disease

**DOI:** 10.4061/2011/716859

**Published:** 2011-06-03

**Authors:** Anna Zinger, Carlos Barcia, Maria Trinidad Herrero, Gilles J. Guillemin

**Affiliations:** ^1^Department of Pharmacology, School of Medical Sciences, University of New South Wales, Sydney, NSW 2052, Australia; ^2^Experimental and Clinical Neuroscience (NiCE-CIBERNED), Department of Human Anatomy and Psychobiology, School of Medicine, University of Murcia, Murcia, Spain; ^3^St Vincent's Centre for Applied Medical Research, Darlinghurst, NSW 2010, Australia

## Abstract

Parkinson's disease (PD) is a common neurodegenerative disorder characterised by loss of dopaminergic neurons and localized neuroinflammation occurring in the midbrain several years before the actual onset of symptoms. Activated microglia themselves release a large number of inflammatory mediators thus perpetuating neuroinflammation and neurotoxicity. The Kynurenine pathway (KP), the main catabolic pathway for tryptophan, is one of the major regulators of the immune response and may also be implicated in the inflammatory response in parkinsonism. The KP generates several neuroactive compounds and therefore has either a neurotoxic or neuroprotective effect. Several of these molecules produced by microglia can activate the N-methyl-D-aspartate (NMDA) receptor-signalling pathway, leading to an excitotoxic response. Previous studies have shown that NMDA antagonists can ease symptoms and exert a neuroprotective effect in PD both *in vivo* and *in vitro*. There are to date several lines of evidence linking some of the KP intermediates and the neuropathogenesis of PD. Moreover, it is likely that pharmacological modulation of the KP will represent a new therapeutic strategy for PD.

## 1. Introduction

Parkinson's disease (PD) is the most common movement disorder and is the second most common chronic progressive neurodegenerative disorder after Alzheimer's disease. PD is a sporadic and age-dependent disease in 90% of cases and affects more than 1% of the world population over the age of 65 [1]. PD is characterised by motor symptoms including bradykinesia, tremor, rigidity, postural instability as well as nonmotor symptoms such as dementia, sleep disturbance, neurobehavioral, and sensory abnormalities [2].

PD is neuropathologically characterized by the loss of midbrain-pigmented neurons in the *substantia nigra pars compacta* (SNpc). Under normal conditions, these neurons produce dopamine at the striatum and other basal ganglia nuclei [3]. It has been estimated that at the onset of PD symptoms, up to 70% of dopaminergic neurons have been lost. Postmortem examinations have also shown that more than 90% of these neurons have been depleted [4]. Dopaminergic loss leads to an irreversible degeneration of the nigrostriatal pathway, followed by stratial dopaminergic denervation which causes pathological changes in neurotransmission of basal ganglia motor circuit and results in characteristic Parkinsonian symptoms [5]. Another pathological hallmark of the disease is the presence of protein inclusions called Lewy bodies (LBs), which are abnormal intracellular *α*-synuclein (SYN) aggregates in the cytoplasm and axons of the remaining neurons [6]. Neurons containing LBs undergo neurodegenerative processes and subsequently die.

To date, there is no available cure for PD. However, L-Dopa and dopaminergic agonists are useful in treating PD symptoms. This type of therapy mainly aims to replace dopamine in the striatum but does not slow neurodegenerative processes. Moreover, long-term use is associated with serious side effects such as dyskinesia and motor fluctuations [7] resulting in a diminished effect of treatment [8]. 

Although the aetiology of PD is relatively unknown, it has been suggested that there is an association with mitochondrial dysfunction in nigral neurons and neurotoxicity from excess glutamate and reactive oxygen species (ROS) production [9, 10]. Microglia are the prime immune cells of the central nervous system (CNS) and are important producers of neuroactive molecules involved in oxidative stress, excitotoxicity and neuroinflammation. Microglia respond to a wide range of immunologic stimuli or CNS injuries and either initiate protective and/or neuroinflammatory processes [11]. The SN contains the highest concentration of microglia compared with other brain areas [12]. 

Resting microglia have a characteristic ramified morphology; the small cell body remains stationary whilst the long branches are constantly moving and are sensitive to any minor physiological changes [13, 14]. At the site of inflammation, activated microglia change their morphology becoming amoeboid and may act similarly to macrophages: they possibly perform phagocytosis, express increased levels of major histocompatibility complex (MHC) antigens, and secrete various cytotoxins, which may ultimately activate additional microglia to remove harmful stimuli and even initiate healing processes [15, 16]. The total number of MHC class II microglia has been shown to be significantly increased not only in SN and putamen but also in the hippocampus, transentorhinal cortex, cingulate cortex, and temporal cortex in PD brains [17]. This implies that microglia are activated and are likely to be associated with the neuropathological phenomenon, which ultimately damages neurons [17, 18]. The microglial reaction is a very tightly regulated process which is essential for a precise immune response; excessive microglial activation leads to a continuous release of inflammatory mediators such as cytokines, chemokines, reactive free radicals, and proteases [19]. This process is referred to as “reactive microgliosis” and involves the proliferation, recruitment, and activation of microglia which is then followed by neuronal damage [20], all of which are secondary to actual neuronal injury. Thus, initial, acute damage from microgliosis may provoke a continuous cycle of events, which then develops into chronic, progressive neurodegeneration which is a common characteristic of Parkinson's disease [21].

## 2. The Role of Neuroinflammation in the Pathogenesis of PD

A large number of studies involving cells, animal models, and human patients indicate the involvement of neuroinflammation in the neuropathogenesis of PD. 

### 2.1. *In Vitro*/*In Vivo*

To demonstrate the delayed and progressive nature of neuroinflammation observed in PD, lipopolysaccharide (LPS) was administered to rodents as a single dose or a chronic infusion [22]. While LPS has no direct effect on neurons, it is capable of initiating a chronic inflammation and a delayed, secondary progressive degeneration of dopaminergic neurons in the SN [22, 23]. An *in vitro* study has also shown that 1-methyl-4-phenyl-1,2,3,6-tetrahydropyridine (MPTP) can initiate direct neuronal injury in neuron-glia cultures which is then followed by the induction of reactive microgliosis [24]. Furthermore, in a microglia free neuronal-astrocytic coculture, MPTP induced only acute, non-progressive neurotoxicity [21]. MPTP is selectively toxic to dopaminergic neurons and is often used to induce an *in vivo* PD-like disease in animal models [25]. Moreover, inhibition of microglial activation results in a strong decrease in neurotoxicity in both MPTP mouse and LPS rat models [26, 27].

### 2.2. Human Studies

A large epidemiological study on approximately 150,000 men and women has shown that the use of nonsteroidal anti-inflammatory drugs (NSAIDs) can prevent or delay the onset of PD [28]. Chen et al. have also observed a similar effect in chronic users of ibuprofen, a NSAID acting on cyclooxygenase (COX) [29]. A correlation has also been found between high plasma concentrations of interleukin-6, a proinflammatory cytokine, and an increased risk of developing PD [30]. Moreover, *in vivo* imaging studies on patients with idiopathic PD have shown an increase in neuroinflammatory areas in basal ganglia, striatum, and frontal and temporal cortical regions compared with age-matched healthy controls [31]. All of these studies suggest that microglial activation occurs at an early stage of the disease either before (or in parallel with) the important loss of dopaminergic neurons. In postmortem PD tissues, activated microglial cells have been detected around impaired dopaminergic neurons in the SN, thus demonstrating the presence of neuroinflammation [32]. As previously discussed, MPTP causes Parkinsonism in both humans and primates. This leads to the chronic presence of activated microglia around dopaminergic neurons in the SN for up to 10 years after exposure [33, 34], even without L-DOPA treatment [35]. Substantial evidence of microglial activation associated with dopaminergic neuronal damage suggests that degenerating neurons initiate microgliosis, which then leads to further neuronal loss. Microglial activation represents an initiator and/or a secondary responder in this disease process. Therefore, suppressing neuroinflammation by preventing microglial activation could potentially slow down or stop this continuous and deleterious cycle which damages neurons.

However, the initial stimulus driving excessive inflammation is still unknown. There are several compounds released by damaged neurons, which are able to induce microgliosis and ROS production. These include (i) matrix metalloproteinase 3 (released by damaged dopaminergic neurons), which induces superoxide production by microglia leading to neuronal death [36]. (ii) Neuromelanin, a neuronal pigment released in PD by dying neurons which is capable of activating microglia [37]. (iii) SYN, a component of LB neurons, typically found in PD that is toxic to neurons but only in the presence of microglia. (iv) Aggregated SYN-activated microglia are toxic to dopaminergic neurons isolated from embryonic mouse brain. Importantly, its toxicity is dependant on the presence of nicotinamide adenine dinucleotide phosphate (NADPH) oxidase following ROS formation [38]. Another study has shown that neuroinflammation is accompanied by dopaminergic loss and aggregation of oxidized SYN in the cytoplasm of SN neurons when human SYN is present in the mouse brain [39]. Taken together, these studies suggest that there is a link between protein aggregation and the production of ROS by activated microglia. 

Over production of ROS by microglia has been directly linked to neuronal toxicity and death via the nitric oxide (NO) mechanism [40, 41]. NO induces oxidative stress, a major cause of neuronal injury, which is strongly linked to the pathogenesis of PD and physiological aging [42, 43]. For example, NO can react with dopamine to generate quinone products, which are known to have a damaging effect on brain mitochondria [44]. Basal level of lipid peroxidation is increased in the SN of PD patients, suggesting a higher sensitivity of this area to free radicals and ROS [45]. Aging also contributes to microglial “priming”: activated microglia in healthy aged brains release excessive quantities of proinflammatory cytokines compared to younger individuals [46, 47]. Furthermore, there is an increased probability of developing a neurodegenerative disorder after 60 years of age due to age-related increases in oxidative, metabolic, or inflammatory activation [48].

Inflammatory cytokines (IL-1*β*, TNF-*α*, IL-6, and IFN-*γ*) are also released by activated microglia and amplify the inflammatory response. Excessive production of these cytokines has been reported in the SN of PD patients [49, 50] as well as in cerebrospinal fluid (CSF) and blood compartments [51, 52]. Cytokines can stimulate inactivated microglia and also directly bind to receptors on the cellular surface of dopaminergic neurons thereby promoting apoptotic cell death and subsequent phagocytosis of DA neurons [53]. Neurons in the midbrain, unlike those in the hippocampus or cortex, exhibit a greater sensitivity to proinflammatory cytokines. Moreover, this sensitivity has been directly related to a high degree of oxidative processes [19]. In contrast, activated microglia also produce anti-inflammatory cytokines such as TGF-*β*1, IL-10, and IL-1. These cytokines play a role in the inhibition of the inflammatory response. Importantly, the balance between pro- and anti-inflammatory cytokine production is impaired during neuroinflammation [54]. 

**Figure 1:**
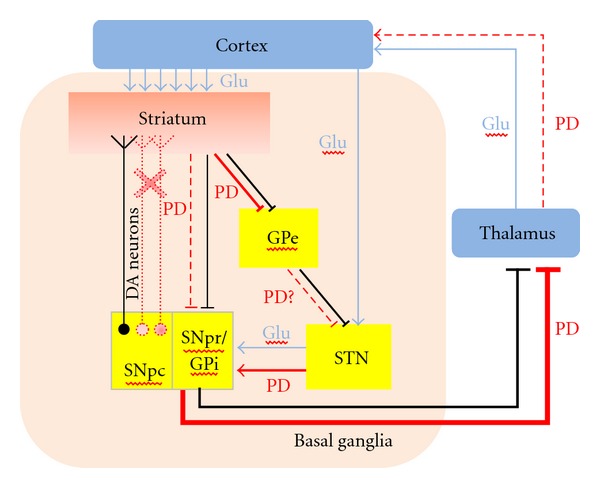
Basal ganglia motor circuit in Parkinson's disease: dopaminergic neurons (DA) create a direct pathway between Substantia Nigra pars compacta (SNpc) and striatum—the input nuclei of the basal ganglia. Another direct pathway connects the striatum to the internal segment of globus pallidus (GPi) and the substantia nigra pars reticulata (SNpr). GPi and SNpr are the output nuclei of the basal ganglia, which projects to the thalamus and from there to the cortex. The indirect pathway connects the striatum to output nuclei through external segment of the globus pallidus (GPe) and then subthalamic nucleus (STN). In Parkinson's disease (PD), the dopaminergic input from SNpc is progressively lost, causing a reduction in the direct pathway signal. Indirect pathway increases its activity through STN in the output nuclei and has inhibitory influence on the thalamus. It leads to a reduction of thalamic glutamateric input on the motor cortex and subsequent reduction in movement, as rigidity and bradykinesia are observed in PD patients.

On the other hand, the excitatory neurotransmitter glutamate plays a critical role in glutamatergic transmission in basal ganglia functions [55]. The action of glutamate on neurons is mediated by ionotropic and metabotropic glutamate receptors. Ionotropic N-methyl-D-aspartate (NMDA) receptors are known to mediate excitotoxicity caused by high levels of glutamate and can be found on dopaminergic neurons [56]. Activation of NMDA receptors located on DA neurons leads to neurotoxicity both *in vitro* and *in vivo* [57, 58]. The functional organisation of basal ganglia also contributes to the genesis of symptoms observed in movement disorder. The striatum (the input nucleus of the basal ganglia circuit) is the main recipient of dopaminergic fibres from the SN. The reduction in dopaminergic innervations of the striatum and changes in the activity of basal ganglia induces complex changes in the structure and function of basal ganglia NMDA receptor [59]. Glutamatergic excitation is increased and glutamatergic neurons become uninhibited under PD conditions, especially due to the excessive firing from the subthalamic nucleus to the SN [60] (Figure 1). It has been shown that the neurotoxicity of activated microglia is primarily mediated by glutamate released through NMDA receptor signalling [61]. Neuritic beading (a focal bead-like swelling in dendrites and axons) is a neuropathological sign in PD [62]. It can also be induced by microglia activated through the NMDA receptor [61]. NMDA receptors have been linked with disturbed energy metabolism and glutamate transmission leading to neuronal death, and have therefore been investigated as important therapeutic targets in pharmacological PD research [63]. Accordingly, reducing glutamatergic transmission may lead to an “anti-PD activity”. Indeed, injections of the NMDA antagonist, MK-801, reverses parkinsonian symptoms in MPTP-treated monkeys [64]. Several studies using rodent PD models have shown that glutamate antagonists have both symptomatic and neuroprotective effects in PD [59]. Recently, PD patients treated with memantine, another NMDA receptor antagonist have shown moderate but significant improvements in terms of cognitive symptoms [65]. The use of amantadine as an adjuvant to levodopa has demonstrated beneficial effects on motor response complications [66]. Additional evidence has been reviewed and has demonstrated the potential of NMDA receptor blockade in reversing parkinsonian symptoms [59].

## 3. The Kynurenine Pathway

**Figure 2:**
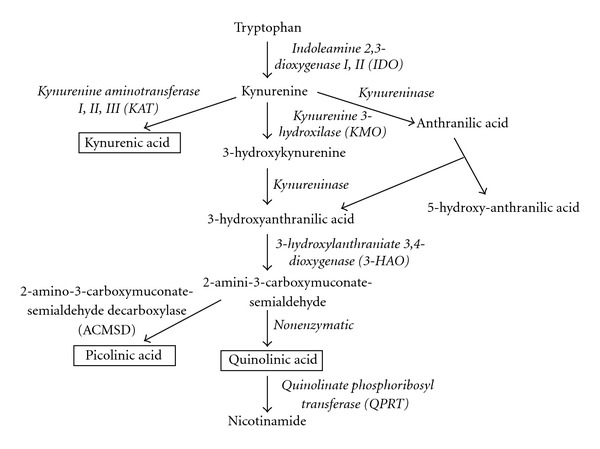
Simplified diagram of Kynurenine pathway: during neuroinflammation, 95% of the dietary tryptophan is metabolized along the KP within the brain. The remaining 5% serves as a precursor to the synthesis of the neurotransmitter serotonin. IDO catalyses the initial and rate-limiting step in the degradation of tryptophan through KP that ultimately leads to the production of nicotinamide.

The kynurenine pathway (KP) represents the main catabolic pathway of the essential amino acid tryptophan (TRP), which ultimately leads to the production of the central metabolic cofactor, nicotinamide adenine dinucleotide (NAD^+^) (Figure 2). The KP is also one of the major regulatory mechanisms of the immune response [67]. Two nonmutually exclusive theories have been proposed: (1) that TRP degradation suppresses T-cell proliferation by dramatically depleting the supply of this critical amino acid and (2) that various downstream KP metabolites suppress certain immune cells [67]. Induction of the KP by the rate-limiting enzyme, indoleamine 2,3 dioxygenase (IDO1) in dendritic cells completely inhibits clonal expansion of T cells [68]. Moreover, TRP depletion and IDO1/KP activation have been implicated in the facilitation of immune tolerance associated with pregnancy and tumour persistence [69]. 

The cellular expression of the KP in the brain is only partially understood. It is complete in cells of monocytic lineage, including macrophages and microglia [70], but only partially present in human astrocytes [71], neurons [72], and endothelial cells [73]. The various KP metabolites can have either neurotoxic or neuroprotective effects and occasionally both depending on their concentration. The neurotoxicity of several KP metabolites has been investigated in relation to oxidative stress generation and neuronal death *in vitro* and *in vivo* in animal models of neurodegenerative disorders [74–77]. 3-hydroxykynurenine (3-HK), 3-hydroxyanthranilic acid (3HAA) and 5-hydroxyanthranilic acid (5HAA) are known to induce cell death in cultures of rat neurons [78]. 3-HK is toxic to stratial neuronal cultures, mainly due to its ability to generate ROS and initiate apoptosis [79]. Quinolinic acid (QUIN) however, is likely to be the most important in terms of biological activity. QUIN can selectively activate NMDA receptors producing excitation and which ultimately causes selective neuronal lesions in the rat brain [80, 81]. Acute QUIN production can lead to human neuronal death and chronic production causes dysfunction by at least six separate mechanisms [82, 83]. In pathophysiological concentrations, QUIN activates the NMDA receptor [84]. QUIN also increases glutamate release in neurons and inhibits glutamate uptake and catabolism in astrocytes. QUIN can potentiate its own toxicity and that of other excitotoxins, for example, NMDA and glutamate thus producing progressive mitochondrial dysfunction [85]. Finally, QUIN can increase free radical generation by inducing nitric oxide synthase production (NOS) in astrocytes and neurons which in turn leads to oxidative stress [86, 87]. Within the brain, QUIN is produced by activated microglia and infiltrating macrophages [70]. Neurons and astrocytes do not produce QUIN [88, 89]. Recent findings have demonstrated that QUIN excitotoxicity in human astrocytes and neurons is mediated through activation of an NMDA-like receptor [87]. In addition, QUIN-induced damage can be increased in the presence of 3-HK, 6-hydroxidopamine, a specific dopaminergic neuron toxin, or ROS [90–92]. Human glial cells, such as astrocytes and microglia produce most components of the KP [93]. The KP components are also present in macrophages that are capable of penetrating the blood-brain barrier (BBB) in the presence of brain damage or infection [94]. Thus, up-regulation of QUIN production alone or with additional neurotoxic factors during inflammation could easily lead to over activation of the NMDA receptor. This is followed by oxidative stress, which occurs in early PD development.

In contrast to the neurotoxic activity of QUIN, kynurenic acid (KYNA) is a neuroprotective metabolite, antagonising all ionotropic glutamate receptors (including NMDA) and thus blocks some of the neurotoxic effects of QUIN and other excitotoxins. KYNA is produced from kynurenine by the kynurenine aminotransferase enzymes (KAT) I, KAT II, and KAT III, in astrocytes [71]. Endogenous generation of KYNA in rat brain has been shown to be more effective than KYNA applied exogenously, suggesting the importance of localised KYNA production and physical proximity to NMDA receptors [95]. An increase in endogenous KYNA levels can prevent SN dopaminergic loss caused by focal infusion of QUIN or NMDA [96]. Nanomolar concentrations of KYNA significantly reduce glutamate output from striatal neurons in rat brain, similar to the kynurenine hydroxylase (KMO) inhibitors [97]. Both, KYNA and QUIN are produced in the SN or the adjoining striatum region [98, 99]. Based on previous studies, it can be hypothesised that under normal conditions local concentrations of KYNA and QUIN are low and physiologically regulate NMDA receptor function. However, in disease states, where QUIN production is high, it is thought that there is insufficient KYNA concentration to block QUIN production [100]. 

Picolinic acid is another endogenous neuroprotective compound [101] and is also the main metal chelator in the brain [102]. Previously, we have shown that it is produced in micromolar concentrations by human primary neurons [72]. PD is associated with neuropathological features such as protein aggregation and oxidative stress associated with the involvement of metal ions [103]. Therefore, use of chelating agents has also been suggested as a form of therapy for PD. 

The KP, under normal physiological conditions is well balanced and produces all KP intermediates leading ultimately to NAD^+^  production. However, under pathologic conditions, IDO1 is activated and astrocytes produce kynurenine (KYN) and KYNA, [104], neurons produce PIC [88] and activated microglia/infiltrating macrophages produce QUIN [89]. It is important to note that PIC and KYNA can partly antagonise the neurotoxic effects of QUIN [105]. However, astroglial secretion of large quantities of KYN can lead to further synthesis of QUIN by microglia, suggesting that the cerebral synthesis of QUIN largely overtakes the neuroprotective effects of PIC and KYNA [106].

## 4. Evidence for the Involvement of the KP in PD

Impaired KP metabolism and altered KYNA levels have been previously reported in the brain of PD patients. This occurs when the KYNA/TRP ratio in serum and cerebrospinal fluid (CSF) is significantly increased together with 3-HK levels, a neurotoxic compound that contributes to oxidative damage in the putamen and SNpc [107, 108]. These findings suggest that endogenous KYNA concentrations are decreased and unable to effectively block NMDA receptor and prevent neurotoxicity induced by 3-HK. KAT I expression, the KP enzyme which leads to KYNA formation, is decreased in the SNpc of MPTP-treated mice [109]. KAT-I immunoreactivity in dopaminergic neurons and surrounding microglia has been linked to increased vulnerability of SN neurons to toxicity. Lowered KYNA concentrations have also been found in the frontal cortex, putamen, and SNpc of PD patients [107]. KYNA, but not the highly selective NMDA antagonist 7-chlorokynurenic acid exhibits partial protection against MPP+ toxin on dopaminergic terminals of rat striatum [110].

However, increased KAT II activity, which is an enzyme responsible for 75% of the KYNA synthesis in the brain, has been found in peripheral red blood cells of PD patients. It is not however found in plasma [111]. The increased KAT II activity correlates with higher blood KYNA concentrations; this elevation may be caused by 3-HK released from the CNS. As KYNA has limited abilities to cross the BBB, it has been suggested that peripheral KYNA is likely to be transported to the brain by large neutral amino acid carriers and there it has neuroprotective effects [112]. Another recent study has shown that KYNA is involved in leukocyte recruitment and the investigators hypothesised that KYNA might therefore have an anti-inflammatory action [113]. Based on preclinical and clinical data, KYNA or its analogues are thought to have neuroprotective effects in PD trough binding as antagonists to the NMDA receptor. This in turn causes slow neuronal excitotoxic damage [114].

**Figure 3:**
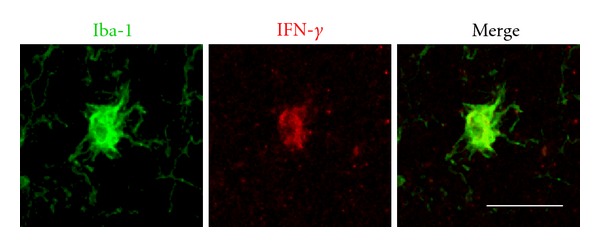
Activated microglial cells express IFN-*γ* in Parkinsonism: confocal images of the immunofluorescence of IFN-*γ* (red) combined with microglia cells marker—Iba-1 (green) in the SNpc of a parkinsonian monkey. Scale bar: 35 mm.

**Figure 4:**
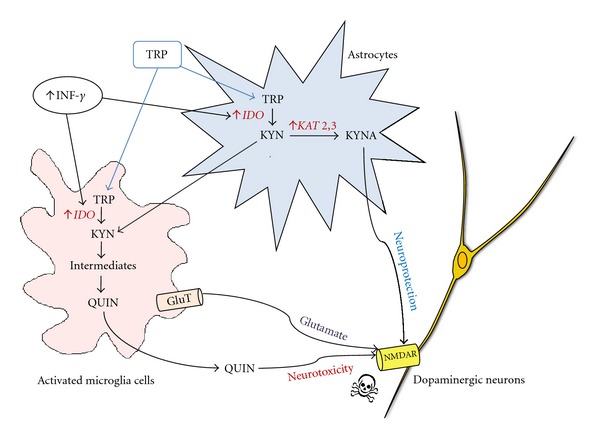
Model for Kynurenine pathway interactions between astrocytes, neurons, and microglia during brain inflammation. Abbreviations: TRP: tryptophan; IDO: Indoleamine 2,3-dioxygenase; KYN: kynurenine: QUIN: quinolinic acid; NMDAR: NMDA receptor; KAT: Kynurenine aminotransferase; GluT: glutamate transporter.

Unpublished data from our group shows an increase in the production of IFN-*γ* by microglia in the SN of MPTP-treated macaques' brain (Figure 3). This is of particular significance, as IFN-*γ* is also a potent inducer of the KP [115]. In the same study, we have also shown that QUIN is produced and accumulated by activated microglia. These microglia colocalise with dopaminergic neurons in the SN of MPTP-treated macaques. Several other studies have shown extensive evidence of activated microglial cells and NMDAR^+^ dopaminergic neurons in the SNpc. This suggests that the NMDA receptor is likely to be activated by endogenous QUIN released by microglia and followed closely by glutamate [116, 117] (Figure 4).

## 5. Recent KP Inhibitors for the Treatment of PD

Several drugs that block the KP are currently under therapeutic investigation both in our laboratory and by other investigators. For example, 4-chlorokynurenine crosses the BBB and blocks QUIN toxicity at the glycine site on NMDA receptors [118]. Kynurenic acid analogues are currently due to enter clinical trials for the treatment of epilepsy, stroke, and possibly PD as potential neuroprotective agents [119]. Two KP analogues are at present under investigation in a phase III clinical trial. These are Teriflunomide (Sanofi-Aventis) and Laquinimod (Teva Neuroscience) [120]. Recently, one KP analogue reached the Japanese market as a potent immunomodulatory drug for the treatment of arthritis, asthma, and dermatitis [120]: Tranilast/Rizaben (Kissei Pharmaceutics) is an anthranilic acid derivative and it has been proposed as a treatment for autoimmune diseases such as Multiple Sclerosis [121]. Finally, 8-OH Quinolinone metal attenuating compounds—Clioquinol and PBT2 (Prana) rapidly decrease soluble brain amyloid-beta and improve cognitive performance [122]. Interestingly, these 2 compounds share close structural similarity and similar biochemical properties with KYNA and QUIN.

Conjugates of KYNA analogues with D-glucose or D-galactose increase its ability to cross the BBB and prevent excitotoxicity and seizures in an animal model [123]. Kynurenine 3-hydroxylase inhibitors significantly reduce the severity of dystonia in hamsters and may therefore be a potential candidate for managing dyskinesia associated with striatal dysfunction [124]. There is also an increasing interest in the use of pharmacological modulation of the KP in treating numerous disorders like AIDS-dementia and many other neurodegenerative diseases, diabetes, depression, infections, tumour development, glaucoma, and cataract formation [116].

## 6. Conclusions

**Figure 5:**
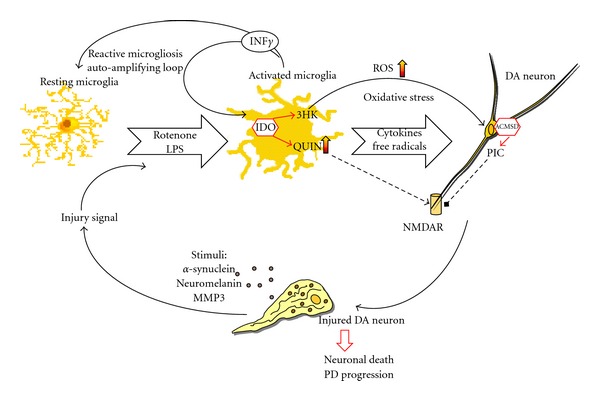
The possible role of Kynurenine pathway involvement in dopaminergic neurodegenerative process through microglia activation: Parkinson's disease is associated with chronic activation of microglia, which also can be induced by LPS or Rotenone treatments. Classic microglia activation release neurotoxic substances including reactive oxygen species (ROS) and proinflammatory cytokines as INF-*γ*, potent activator of Kynurenine pathway (KP). KP in activated microglia leads to upregulation of 3HK and QUIN. 3HK is toxic primarily as a result of conversion to ROS. The combined effects of ROS and NMDA receptor-mediated excitotoxicity by QUIN contribute to the dysfunction of neurons and their death. However, picolinic acid (PIC) produced through KP activation in neurons, has the ability to protect neurons against QUIN-induced neurotoxicity, being NMDA agonist. Microglia can become overactivated, by proinflammatory mediators and stimuli from dying neurons and cause perpetuating cycle of further microglia activation microgliosis. Excessive microgliosis will cause neurotoxicity to neighbouring neurons and resulting in neuronal death, contributing to progression of Parkinson's disease.

PD seems to be associated with an imbalance between the two main branches of the KP within the brain. KYNA synthesis by astrocytes is decreased and concomitantly, QUIN production by microglia is increased (Figure 5). There are many therapeutic opportunities for intervention and modification of an impaired KP that may prevent the progression of neurodegenerative disorders such as PD. Using specific KP enzyme inhibitors, it may be possible to reinstate a physiologically normal KP, which is neuroprotective. This neuroprotective state might also be synergistically improved by concomitantly blocking the NMDA receptor using its antagonists, such as memantine or MK801. Additionally, neuroprotection may be achieved by designing KYNA analogues that are able to penetrate the BBB and deliver neuroprotective compounds to brain pools thus reducing hyperactivation of glutamatergic receptors.
